# Functional and subjective outcomes after surgical management of complex elbow dislocations: a retrospective study

**DOI:** 10.1007/s00590-024-04103-5

**Published:** 2024-09-20

**Authors:** Riccardo Giai Via, Carlotta Faccenda, Stefano Artiaco, Elisa Dutto, Alessandro Dario Lavia, Alessandro Massè, Bruno Battiston

**Affiliations:** 1 Department of Orthopaedic Surgery, Centro Traumatologico Ortopedico (CTO), University of Turin, Via Gianfranco Zuretti, 29, 10126 Turin, Italy; 2 Hand Surgery Department, Ospedale San Paolo, Via Genova 30, 17100 Savona, Italy; 3 Centro Traumatologico Ortopedico (CTO), UOC Hand Surgery and Microsurgery Reference Centre Piedmont Region, Via Gianfranco Zuretti, 29, 10126 Turin, Italy; 4 Department of Economics, Boston College, Boston, MA USA; 5 Department of Orthopaedics and Traumatology, CTO, University of Turin, Via Zuretti 29, 10126 Turin, Italy

**Keywords:** Elbow dislocations, Complex dislocations, Complex elbow dislocations, Elbow ligament lesions, Radial head fractures, Coronoid fractures

## Abstract

**Introduction:**

Dislocations of the elbow are the second most frequent upper-body injury after shoulder dislocations, comprising 11–28% of all elbow injuries. Complex elbow dislocations pose challenging management due to the involvement of critical stabilizing structures. This study aimed to investigate functional and subjective outcomes (MEPS, DASH, Oxford score) in 44 patients with complex elbow dislocations who underwent surgery between 2018 and 2020, with subgroup analysis focusing on gender and age differences.

**Material and methods:**

A retrospective analysis was conducted on patients treated at C.T.O. Hospital, Turin, for complex elbow dislocations between January 2018 and December 2020. Surgical approaches included radial head synthesis, coronoid fixation, ligamentous repair, and ulnar nerve management. Postoperatively, patients followed a standardized or individualized program. Data analysis involved t-tests to assess score differences between subgroups.

**Results:**

Among the 44 analyzed patients, the mean age was 48 years, and the mean follow-up time was 29 months. Various types of complex dislocations were identified, with radial head and coronoid fractures classified accordingly. Surgical approaches included multiple methods of reduction and synthesis. While most patients adhered to postoperative programs, subsequent reoperations were conducted in 11% of cases. Scores did not significantly differ between genders, although a trend toward better DASH scores in males was observed. Younger patients showed better, though not statistically significant, outcomes in mobility and functional measures.

**Conclusion:**

This study underscores the importance of pre-operative assessment for positive surgical outcomes in complex elbow dislocations. Additionally, findings suggest that younger age may correlate with slightly better functional outcomes. Despite limitations such as retrospective design and sample size, the study enhances our understanding of complex dislocation outcomes and provides valuable insights for future interventions.

## Introduction

Dislocations of the elbow are the second most frequent in the upper body after the shoulder, accounting for 11–28% of total elbow injuries [1–3].

Elbow dislocations can be classified as simple or complex based on the presence of associated bone fractures. These account for about 25% of total dislocations of the elbow [4]. Complex dislocations are difficult-to-manage injuries that can bring rigidity, residual instability, or pain to a greater extent than simple dislocations since important stabilizing structures of the elbow are more often injured, including bony constraints (such as the coronoid) and soft tissue constraints [5]. Elbow dislocations can present differently depending on the direction and duration of the forces applied during the injury. The progression of elbow injuries typically starts from the lateral side and moves medially, with the first failure being the Lateral Collateral Ligament (LCL) and the last being the Medial Collateral Ligament (MCL) [6]. In complex elbow dislocations, four patterns of fracture-dislocation are described: posteromedial rotatory instability (PMRI) [7, 8], posterolateral rotatory (also known as terrible triad) [7, 9], Monteggia fracture-dislocation [10] or variants in which an axial and bending force is applied at the proximal forearm. The latter can be further divided into anterior apex lesions with an intact radial head or posterior apex lesions with an associated radial head fracture [11]. Accurate clinical and radiographic investigation to determine the exact bone and soft tissue damage involved in this type of elbow injury is crucial to allow a more specific surgical approach for the type of damage to have more satisfying postoperative functional outcomes [12].

The main objective of this study is to show the functional and subjective outcomes (MEPS, DASH, Oxford score) of 44 patients with complex elbow dislocation who had surgery at a Level I orthopedic center between 2018 and 2020. We further divided our patient sample into women and men to see if the latter, who are generally more prone to high-energy trauma [13], had fewer outcomes, and young and elderly to see if age influenced the final outcome [14].

## Material and methods

This retrospective study analyzed surgically treated patients with complex elbow dislocations treated surgically at C.T.O. Hospital in Turin. The period included runs from January 2018 to December 2020.

The study included only patients who underwent surgery for complex elbow dislocation and were at least 18 years old.

During the time interval examined, 54 patients underwent surgery, ten did not reply to the call and/or were unavailable to attend a new clinical evaluation, and one of whom died of unrelated causes.

A total of 44 patients agreed to undergo clinical re-evaluation.

All the patients were admitted to our Level I DEA center between 2018 and 2020. Our trauma center is the principal of two Level I DEAs in our metropolitan district. After a brief history taking on the nature and etiology of the injury and any influential remote history, an X-ray is always performed before and after a reduction in narcosis, then the stability in varus-valgus stress is checked, and the elbow is immobilized with a posterior valve cast. A follow-up CT scan is then performed using the ‘‘Span—Scan—Plan’’ scheme.

After surgery, each patient was hospitalized for two days and discharged home if clinical conditions were stable and if the postoperative x-rays satisfied the surgeon. Patient-specific indications and clinical and radiographic follow-ups (usually performed at 30,60 and 90 days postoperatively) are given in the discharge letter. Depending on the scenario identified at the outpatient visit, rehabilitation indications were given at outpatient follow-ups.

Our study reviewed patient demographics and the waiting time between injury and surgery. Through the evaluation of the pre-operative X-ray, CT scan and the re-evaluation of intra-operative reports, the initial injuries were divided into different types of fracture-dislocation: terrible triad, trans-olecranon, Monteggia-like and ‘‘other’’ when it was not possible to place it in a specific category.

In addition, we classified radial head fractures according to Hotchkiss classification and coronoid fractures according to O’Driscoll’s classification.

The patient’s and surgical records were evaluated, as were surgical access, type of synthesis, and accessory surgical gestures such as the opening of the tunnel cubital and anteposition of the ulnar nerve, history of nerve suffering, and rate of re-interventions.

During the outpatient visit, the patient’s range of motion (ROM) was tested for both flexion–extension (FE) and pronation-supination (PS). Additionally, stability tests were performed to evaluate varus-valgus stress. Patients were also subjected to subjective scores such as the Disabilities of the Arm, Shoulder and Hand (DASH) score [15], the Oxford Elbow Score (OES) [16] and the Mayo Elbow Performance Score (MEPS) [17]. In addition, the patient’s history of nerve injury symptoms was reviewed, and a new clinical evaluation was conducted. Tinel’s sign, Froment’s sign, and Wartenberg’s sign were all used to assess ulnar nerve function at the elbow, and the results were classified using McGowan's classification system. In addition, the last follow-up X-ray evaluated the state of bone healing and the development of heterotopic calcifications. Only patients with unsatisfactory results or significant changes in health status compared to the previous visit received a new X-ray to reduce X-ray exposure.

### Data analysis

In this research study, a t-test was performed to analyze the variations in scores among patients who had undergone complex dislocations. The patients were divided into two subgroups based on their gender (male and female) and age (below 60 years old and above 60 years old). The aim was to investigate whether there were statistically significant variations in the scores, including Meps, Meps pain, Meps mobility, Meps function, DASH, and Oxford. The analysis involved combining the data by group and employing a t-test analysis. It is important to note that the t-test assumed that the variances for the two populations were the same.

A p-value below 0.05 was used as the threshold to reject the null hypothesis, which stated that the difference in means was not statistically significant.

The statistical analysis was performed using STATA software, version 17 (2021).

## Results

In total, of the 44 patients analyzed, 29 were males (66%) and 15 females (34%), while the mean age found was 48 years (16–77). The mean time between injury and surgery was ten days. The mean follow-up time recorded was 29 months (13–48), as shown in Table 1 CT studies identified various complex elbow dislocations: 24 terrible triads (54%), 3 trans-olecranon fracture-dislocations (7%), 4 Monteggia-like fracture-dislocations (9%), and 13 other unclassified complex injuries (30%). Radial head fractures treated were divided according to Hotchkiss' classification, identifying 2 types 1 (4%), 8 types 2 (18%) and 19 types 3 (43%). Coronoid fractures were classified by O'Driscoll classification, with 20 type A (45%), 7 type B (16%) and 5 type C (11%) fractures. In addition, 7 sublime tubercle fractures were identified (16%) (see Table 2).

**Table 1:** Main epidemiologic characteristics of patients analyzed in the study

Patient	Age	Gender	Days from injury to surgery	Follow-up (months)
G.M.L	60	F	13	48
S.F	49	M	8	48
A.S	50	M	14	48
E.A	22	M	22	46
R.P	20	M	N/A	45
F.V	54	M	8	44
C.B	45	M	10	44
I.L.C	33	M	13	44
A.D.G	69	F	8	43
P.L	47	M	10	42
D.P	65	M	7	41
I.T	20	M	9	41
C.F	60	F	14	41
S.S	20	M	13	38
M.R.M	70	F	9	37
F.E.M	46	F	11	36
B.J.N	64	F	17	36
A.M	30	M	N/A	35
P.C	48	M	6	34
A.B	65	F	0	32
F.T	41	M	12	31
S.Z	30	M	9	27
P.M	65	F	13	27
C.I	73	F	17	27
S.A	23	M	9	24
V.Z	63	M	7	22
F.P	56	M	2	20
P.F	69	M	10	19
S.T	41	M	6	19
R.B	61	F	3	19
A.M	54	M	2	18
L.C	16	M	5	18
E.G	57	F	4	18
S.M	18	M	12	16
S.G	53	M	15	16
R.B	35	M	30	16
M.C	52	M	9	16
C.T	71	F	16	16
L.M	68	F	17	15
A.V	26	M	1	14
S.M	60	M	7	14
M.M	53	F	12	14
C.M	77	F	9	14
C.M.P	31	M	12	13

N/A: information not available

**Table 2:** Radial head fractures and coronoid fractures were classified according to Hotchkiss’s and O'Driscoll’s classifications

Patient	Hotchkiss type 1	Hotchkiss type 2	Hotchkiss type 3	O’Driscoll type A	O’Driscoll type B	O’Driscoll type C
G.M.L	1	0	0	–	B3	–
S.F	0	0	0	A2	–	–
A.S	0	0	0	A1	–	–
E.A	0	0	0	–	B1	
R.P	0	0	0	–	–	C1
F.V	0	0	0	A2	–	–
C.B	0	0	0	–	–	–
I.L.C	0	0	0	A2	–	–
A.D.G	0	0	0	–	B2	–
P.L	0	0	0	–	–	C1
D.P	0	0	0	A1	–	–
I.T	0	0	0	–	–	C1
C.F	0	1	1	–	–	–
S.S	1	0	0	–	–	–
M.R.M	0	0	0	–	B3	–
F.E.M	0	0	0	–	B2	–
B.J.N	0	0	0	–	–	–
A.M	0	0	0	–	–	–
P.C	0	0	0	–	–	–
A.B	0	0	0	–	B2	–
F.T	0	0	0	A2	–	–
S.Z	0	0	0	–	B3	–
P.M	0	1	1	–	B3	–
C.I	0	1	1	A2	–	–
S.A	0	0	0	A2	–	
V.Z	0	0	0	A2	–	–
F.P	0	0	0	A2	–	–
P.F	0	0	0	A2	–	–
S.T	0	0	0	A2	–	–
R.B	0	1	1	–	–	C2
A.M	0	1	1	A2	–	–
L.C	0	0	0	–	–	–
E.G	0	1	1	–	–	C2
S.M	0	0	0	–	–	–
S.G	0	0	0	–	–	–
R.B	0	0	0	A2	–	–
M.C	0	0	0	A1	–	–
C.T	0	0	0	A2	–	–
L.M	0	0	0	A1	–	–
A.V	0	0	0	A2	–	–
S.M	0	0	0	A2	–	–
M.M	0	1	1	–	–	–
C.M	0	1	1	A2	–	–
C.M.P	0	0	0	–	–	–

The patients underwent treatment using various surgical approaches. Of the total patients, 23 (52%) were treated with lateral and medial access, and one patient was treated with lateral and posteromedial access. 11 patients (25%) were treated with posterior access, 6 (14%) with lateral access, 3 (7%) with medial access, and 1 patient (2%) with anteromedial access. The average duration of surgery was 155 min (70–255). Different methods of reduction and synthesis were used, as well as various surgical accesses. The radial head was synthesized in 10 patients (23%) with two headless cannulated screws (HCS) (one case with one screw and one with 3 screws, an additional case with one screw combined with a plate). However, radial head synthesis was impossible in 18 patients (41%), requiring prosthetic replacement. Capitellectomy, on the other hand, was not used for any patient. Different methods of synthesis were used for the coronoid: 9 patients (20.5%) were treated with anterograde HCS screw, 7 with anchors (16%), 2 with transosseous sutures (4.5%), 2 with a dedicated plate (4.5%), and 1 patient instead with a retrograde HCS screw.

The olecranon was synthesized in 11 patients (25%) using different methods: the most commonly used was a single plate with screws used in 8 patients (18%), double plate was used in 2 cases (4.5%) (one case with bone autograft and Kirshner wires), and an haubanage was performed in 1 patient (2%).

The lateral ulnar collateral ligament (LUCL) and medial collateral ligament (MCL), in case they were involved in the type of injury and in case they were found during surgery detached or disinserted, were treated surgically. The LUCL was surgically treated in 25 patients (57%): in 23 patients (52%), it was reinserted with a single anchor (only in 2 patients with 2 anchors), and in 2 patients (4.5%), it was repaired with a direct suture. The LCM was treated in 21 patients (48%): 20 patients (45%) with anchor reinsertion(3 patients with double anchors), 1 patient (2%) with direct suture of the LCM. 13 patients (30%) had ligamentous injuries of both medial and lateral compartment.

In some patients, additional surgical procedures were performed alongside ligament stabilization and reduction, such as ulnar nerve anteposition or opening of the cubital tunnel. Ulnar nerve anteposition was performed in 8 patients (18%), with 6 receiving subcutaneous anteposition (14%) and 2 receiving submuscular anteposition (4.5%). Other 8 patients (18%) received cubital tunnel opening with isolated neurolysis.

Regarding the postoperative program, a standardized program was applied for 42 patients, accounting for 95% of the total. The program involved immobilizing the elbow at a 90° angle using a valve cast, followed by replacing it with an articulated elbow brace. Patients were allowed unrestricted prone supination, and gradual release of the brace up to 130°-20° of flexion extension was granted approximately 40 days after the surgery. Two patients (4.5%) received individualized postoperative programs due to immobilization with an external elbow fixator.

Of the 44 patients who had surgery, 5 (11%) underwent subsequent reoperation, while 4 (9%) are awaiting surgery. 4 (9%) out of 5 operated patients had their synthesis devices removed; 3 patients (7%), on the other hand, underwent surgery for ulnar nerve anteposition, and 1 (2%) of these patients underwent coronoid reconstruction with capitulum allograft and capitulum humeri reconstruction with scaphoid allograft; whereas 2 patients(4.5%) underwent arthrolysis (including one anterior and one posterior) for heterotopic calcifications at the elbow. Finally, one patient (2%) underwent reoperation for a surgical wound complication with infectious outcomes post-antibiotic therapy; the patient also underwent ulnar nerve neurolysis and then a third reoperation for a total elbow prosthetic replacement.

All operated patients followed a standard protocol of elbow radiographic follow-ups at 30-60-90 days postoperatively; through these x-rays, it was possible not only to evaluate the outcomes of the synthesis but also to assess heterotopic calcifications of the elbow by using the Ilhai Gabel classification [18]. Thirteen patients with grade I (30%), 8 with grade II (18%) and 5 with grade III (11%) were identified. Two of these patients underwent arthrolysis, one with grade II and the other with grade III. One patient with grade III is still waiting for surgery.

After removing the cast valve and placing the elbow brace with gradual releases, the patient was prescribed physiotherapy to aid in the recovery of the joint. Of the 44 patients who underwent surgery, 95.5% followed the prescribed physiotherapy. Only two patients (4.5%) did not undergo any physiotherapy. We also asked patients about the number of months duration of physical therapy, finding an average of 5.1 months per patient with a minimum of 1 month and a maximum of 12.

For the current study, we called the patients back and conducted an outpatient examination. We performed a thorough and objective examination, documenting the range of motion (ROM) in flexion–extension and prone supination. The results are presented in Table 3. Additionally, we looked for signs of instability at stress in varus-valgus. Only one patient (2%) was found to be unstable at external maneuvers, who had previously undergone treatment with an external fixator and subsequently underwent a second surgery. During the visit, the condition of peripheral nerves, particularly the ulnar nerve, was carefully evaluated: 6 patients (14%) had sporadic dysesthesias in the ulnar nerve region; 10 patients (23%) had signs of ulnar nerve suffering; 5 patients (11.5%) had a positive Tinel's sign at the elbow [19], 2 patients (4.5%) had positive Froment's sign [20] and 2 (4.5%) had positive Wartenberg's sign [21]. Patients undergoing ulnar nerve anteposition were also subjected to McGowan classification [22], which showed 1 patient with grade I (2%), 4 patients with grade II (9%) and 1 patient with grade III (2.5%).

**Table 3:** Objective and subjective scores of the patients cohort included in the study

Patient	ROM FE	ROM PS	MEPS	DASH score	OES
G.M.L	130-0	90-0-90	85	10	N/A
S.F	150-0	90-0-90	100	0.8	46
A.S	130-30	80-0-80	N/A	9.2	41
E.A	150-0	90-0-90	100	3.3	47
R.P	150-0	90-0-90	100	1.7	48
F.V	100-5	05-0-10	95	3.3	47
C.B	150-0	90-0-90	100	2.3	47
I.L.C	130-10	90-0-90	100	0	48
A.D.G	100-40	45-0-45	N/A	N/A	N/A
P.L	110-15	90-0-90	N/A	N/A	N/A
D.P	140-10	60-0-70	85	25.8	28
I.T	130-10	90-0-90	85	24.2	36
C.F	140-15	90-0-90	100	2.5	48
S.S	150-10	90-0-80	100	5.8	47
M.R.M	150-0	90-0-90	100	0	48
F.E.M	50-10	30-0-30	30	77.5	15
B.J.N	140-5	90-0-90	100	3.3	48
A.M	120-10	90-0-20	85	10.8	41
P.C	140-20	80-0-80	85	11.7	43
A.B	150-0	90-0-90	100	12.5	48
F.T	130-15	90-0-80	100	3.6	48
S.Z	150-0	90-0-90	100	0	48
P.M	140-30	80-0-60	65	31.7	27
C.I	140-5	80-0-80	100	10.8	48
S.A	140-10	80-0-80	100	1.7	47
V.Z	90-90	0	45	40.8	26
F.P	150-0	90-0-90	100	0.8	48
P.F	150-0	90-0-50	100	0	48
S.T	140-10	60-0-80	100	4.2	44
R.B	130-30	45-0-70	70	40	24
A.M	140-5	90-0-90	100	0.8	48
L.C	130-10	80-0-80	100	2.5	48
E.G	140-10	90-0-90	100	1.7	45
S.M	140-30	90-0-90	85	23.3	44
S.G	100-80	5-0-5	35	63.8	18
R.B	130-40	30-0-40	75	39.2	26
M.C	130-20	90-0-90	65	37.5	37
C.T	140-10	90-0-90	100	1.7	48
L.M	50-50	0-0 60	55	31	27
A.V	100-10	45-0-40	65	40	24
S.M	150-0	90-0-90	100	0.8	47
M.M	135-30	10-0-20	65	32.5	26
C.M	130-10	80-0-80	95	7.5	40
C.M.P	120-30	80-0-80	85	5	44

FE: Flexion–extension, PS: pronation-supination, ROM: Range Of Motion, MEPS: Mayo Elbow Performance Score, DASH: Disabilities of the Arm, Shoulder and Hand, OES: Oxford Elbow Score N/A: information not available

During the follow-up visit, 42 of the 44 patients (95,5%) included in the study were also evaluated with subjective scores such as Disabilities of the Arm, Shoulder and Hand (DASH) [15], Oxford Elbow Score (OES) [16] and Mayo Elbow Performance Score (MEPS) [17]. The results obtained are best displayed in Table 3; the average of the DASH score obtained was 14 (0-77-5), while for the OES, the average reported was 40.4 (15–48) and finally, for the MEPS, the average identified was 86.7 (30–100).

The patients were divided into different subgroups based on their age and gender. After analyzing the difference between males and females, it was found that none of the scores (MEPS, DASH, and OXFORD) showed any statistically significant difference in means, as depicted in Table 4 This suggests that it was not possible to distinguish between females and males based on these scores and determine which group had better outcomes. However, it is worth noting that the mean DASH score slightly favored males. This observation indicates a trend toward better scores in males, although the difference was not statistically significant.

**Table 4:** Direct comparison of subjective scores between males and females analyzed in the study

	N Male	Mean Male	N Female	Mean Female	*p*-value
MEPS	29.00	88.10	15.00	81.33	0.28
MEPS Pain	29.00	37.24	15.00	36.00	0.71
MEPS Mobility	29.00	18.45	14.00	15.71	0.09
MEPS Function	29.00	22.41	14.00	18.93	0.11
DASH	29.00	13.81	15.00	19.58	0.34
OES	28.00	41.57	13.00	37.85	0.27

N: Number, MEPS: Mayo Elbow Performance Score, DASH: Disabilities of the Arm, Shoulder and Hand, OES: Oxford Elbow Score

In terms of the age comparison between older (> 60 years) and younger patients, the results demonstrated that none of the utilized scores significantly favored the younger patients, as displayed in Table 5 But it’s worth saying that younger patients (< 60 years) showed slightly better results in almost all the subjective scores excluded the DASH score. These findings suggest that younger individuals experienced better mobility and functional outcomes following complex dislocations. However, no clear distinctions were observed among the other scores, indicating that age might not significantly impact those particular measures. Nevertheless, the overall trend suggests that younger patients exhibit better scores across the analyzed measures.

**Table 5:** Direct comparison of subjective scores between young (< 60 years) and elderly (> 60 years) patients analyzed in the study

	N young	Mean young	N elderly	Mean elderly	*p*-value
MEPS	28.00	86.43	16.00	84.69	0.78
MEPS Pain	28.00	36.96	16.00	36.56	0.90
MEPS Mobility	28.00	18.21	15.00	16.33	0.24
MEPS Function	28.00	21.25	15.00	21.33	0.97
DASH	28.00	15.88	16.00	15.59	0.96
OES	27.00	40.78	14.00	39.64	0.74

N: Number, MEPS: Mayo Elbow Performance Score, DASH: Disabilities of the Arm, Shoulder and Hand, OES: Oxford Elbow Score

## Discussion

The most important finding of our study is that a thorough pre-operative assessment and in-depth knowledge of anatomy and trauma biomechanics lead to satisfactory surgical outcomes with good results in range of motion (ROM) in flexion extension and favorable scores in MESH, Oxford and DASH assessments.

Due to the inherent heterogeneity of the pathology, it is not feasible to discuss the specific treatment for each type of lesion in detail. However, comparable treatment strategies were employed based on the specific injury characteristics.

**Fig. 1:**
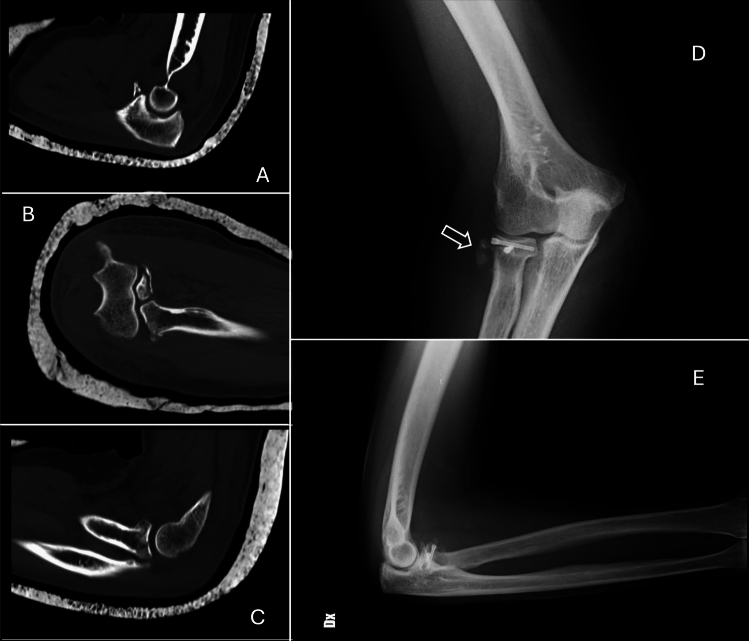
A series of images depicting a patient treated for the terrible triad injury. Images A and B display sagittal and coronal CT scans, respectively, showing a fracture of the coronoid process. Image C presents a sagittal CT scan revealing a fracture of the capitellum. Although the elbow dislocation is part of the terrible triad injury, it is not dislocated in these images but rather stabilized in a cast. Images D and E are postoperative anteroposterior (AP) and lateral radiographs. Note the use of an HCS screw for fixation at the radial capitellum, with heterotopic calcification of the adjacent soft tissues (indicated by the white arrow)

For terrible triad injuries, we used a dual surgical approach (lateral and medial). When possible, radial head fractures were fixed using screws, while more complex cases required radial head prosthesis replacement. Coronoid fractures were managed according to the size of the fragment and degree of comminution: larger fragments were fixed with screws, while comminuted fractures were addressed with a dedicated plate. For very small fragments, transosseous sutures or anchors were used to reattach the capsule (see Fig. 1).

Trans-olecranon fracture-dislocations were treated using a posterior surgical approach. The proximal ulna fracture was stabilized with a single or double proximal ulna plate. In these cases, ligament repair was not always necessary. In one patient with a concurrent radial head fracture, a prosthetic replacement was performed.

Monteggia-like dislocations were all managed with a posterior surgical approach. In cases with an associated proximal ulna fracture, the fracture was stabilized with a plate. One patient with a coronoid fracture was treated with a screw.

**Fig. 2:**
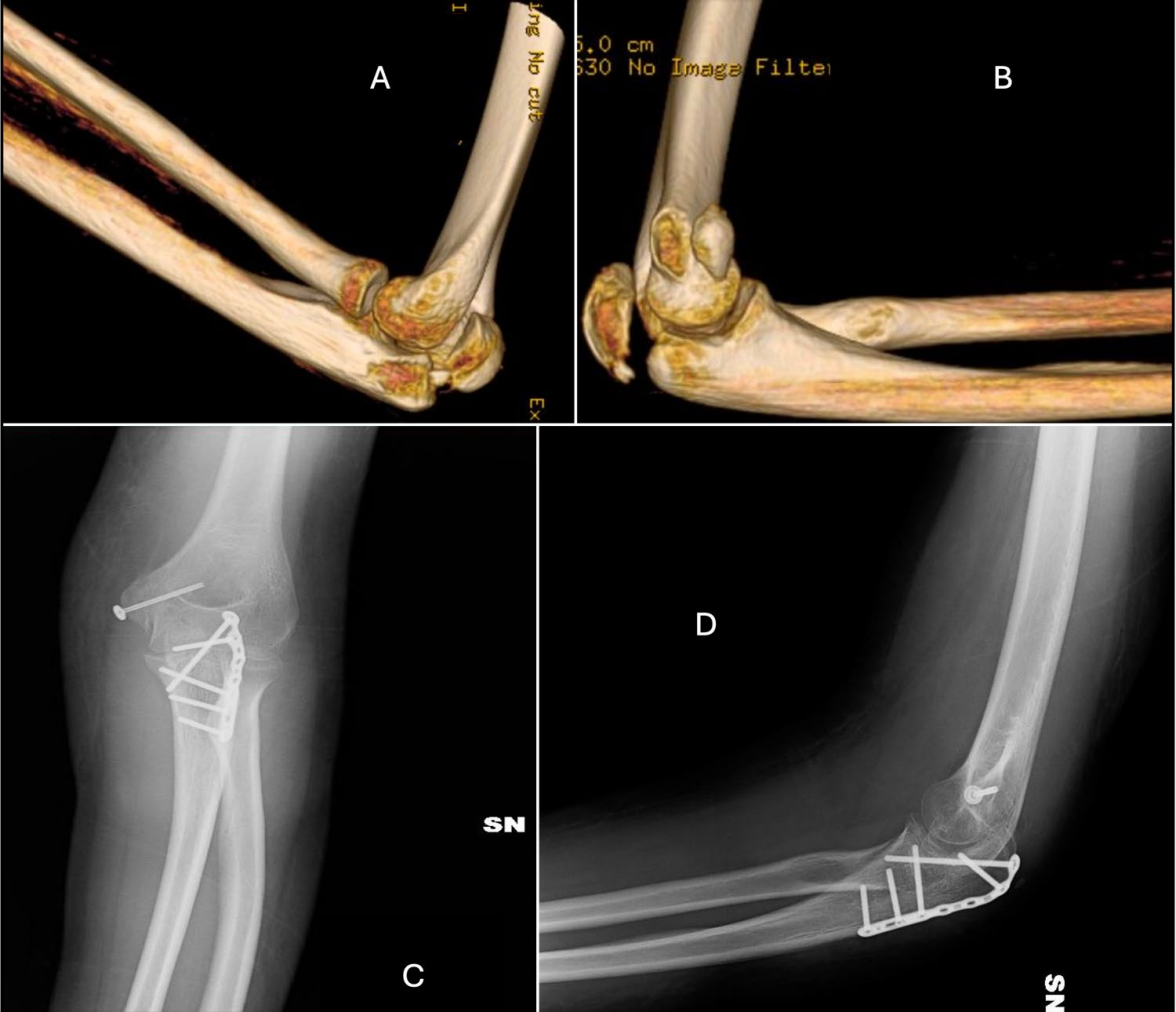
Images A and B display the 3D reconstruction of a complex elbow dislocation with fracture of the olecranon and epitrochlea. Images C and D show postoperative anteroposterior (AP) and lateral X-rays, where the olecranon plate and a screw with a washer for epitrochlear fixation are visible

For the unclassifiable injury group, the treatment principles applied were comparable to those used for the three primary injury types (see Fig. 2).

In all injury types, lesions of the lateral or medial ligament complex were frequently present and treated with direct suturing or reattachment using anchors. However, two patients had residual instability that required external elbow fixation.

Within our patient pool, limitations in flexion ROM were perceived more negatively compared to extension deficits, which were more easily tolerable and had a lesser impact on daily activities. However, among the six patients with restrictions in pronation-supination ROM, the highest dissatisfaction rates were observed. These patients recorded the lowest scores in MESH and Oxford assessments and the highest DASH scores among the entire study population.

Interestingly and importantly, it should be noted that two patients who did not follow the standardized postoperative program like the others but were treated with an individualized postoperative external fixation program still achieved good ROM levels and favorable subjective scores.

Regarding patients who underwent ulnar nerve anterior transposition, they showed lower average scores in MESH (77) and Oxford score (36.8) compared to the overall patient average (86.7 MESH and 40.39 Oxford score). Patients who underwent ulnar nerve neurolysis also exhibited lower average scores in MESH and Oxford assessments (68.3 and 23.6, respectively) but higher DASH scores (34.5). The tendency toward slightly poorer outcomes is likely attributed to the severity of the initial trauma and the potential nerve damage it may have caused. However, the authors have not found any literature describing the outcomes of ulnar nerve anterior transposition in complex elbow dislocations. It is documented that performing more surgical interventions on the nerve tends to lead to more unsatisfactory results [23, 24].

Another interesting aspect to highlight is that among patients who underwent subsequent surgical interventions, excluding those who were re-operated for simple synthesis removal, their ROM was below the study's average, especially in pronation-supination. These patients also reported more unsatisfactory subjective scores, with lower Oxford and MESH scores and higher DASH scores than the average.

In addition, previous studies have demonstrated a specific advantage of the DASH score – it captures psychosocial factors that are less straightforward to define, thereby presenting a more comprehensive representation of subjective outcomes [25]

Similarly, patients with heterotopic calcifications, particularly those classified as grade 3 according to the Ilhai Gabel classification and associated with contracture, showed lower ROM scores with higher degrees of maximum extension and limitations in pronation-supination. As described in several literature articles, heterotopic ossifications can remain asymptomatic but may become symptomatic, limiting ROM, causing contractures, and, in extreme cases, leading to ankylosis [26, 27]. Additionally, peripheral nerves of the upper extremity are susceptible to compression by heterotopic ossification, which can result in clinical symptoms and functional disability [28, 29].

The comparison between young and elderly patients with complex elbow dislocations is also described in the study by Mühlenfeld et al. [30], where the age differentiation is set at 65 years. Interestingly, the mean age observed in the study, which evaluates both simple and complex elbow dislocations, is similar to that of our research, approximately 48 years. Similarly, the male-to-female ratio of 1.9:1 in Mühlenfeld et al.'s study aligns with that identified in our research. Mühlenfeld et al. also noted a younger age among male subjects involved in elbow dislocations, which might be attributed to their propensity for high-energy traumas, as we supposed. It is worth noting that although males represent the majority of the patient pool, they are the clear majority in the under-60 age group, accounting for 25 out of 28 recorded cases. This observation further supports the earlier thesis [13, 30]. Mühlenfeld et al. also showed that adult and elderly patients were as likely to sustain simple or complex elbow dislocations [30].

Regarding functional scores and postoperative range of motion (ROM) recovery, to the authors' knowledge, this represents the first study to individually evaluate such a significant number of complex elbow dislocations, encompassing all four patterns, without being associated with simple dislocations [6–9].

This current study has certain limitations that warrant highlighting. Firstly, the study was conducted retrospectively with its intrinsic limitations. Secondly, we reported a medium-term follow-up with a relatively small sample size of patients. Thirdly, various types of injuries were analyzed and operated on by multiple surgeons using different surgical techniques and fixation methods on a case-by-case basis. The lack of standardized surgical procedures could introduce potential bias. Fourthly, although various scores were proposed to the patients, not all were completed by the entire cohort. Fifthly, the patient’s pool was not further subdivided into subgroups based on fracture types and lesions because we would have had too few patients per category, reducing the statistical validity. With the available data, we aimed to provide a comprehensive understanding of the outcomes of complex elbow dislocations as a whole. However, we acknowledge that future high-quality studies with larger patient pools will be necessary to explore these outcomes more deeply in the specific subgroups of complex elbow dislocation and to reinforce the results obtained in this study.

## Conclusions

When the mechanism of injury is thoroughly studied, and the type of injury is well evaluated preoperatively, patients with complex elbow dislocations can achieve positive outcomes from surgical intervention with good functional results and subjective scores. Moreover, this study did not discover statistically significant differences between females and males concerning the examined scores. Nonetheless, there was a slight trend toward better scores among males, particularly regarding the DASH measure. Additionally, the results indicated that younger patients had better mobility and functional outcomes than older patients, suggesting age-related disparities in these areas. These findings contribute to our comprehension of factors influencing patient outcomes following complex dislocations and may provide insights for future treatment strategies and interventions.

## Data Availability

Dataset analyzed in this study is available from the corresponding author on reasonable request.
